# Profiling HIV Risk and Determined, Resilient, Empowered AIDS-Free, Mentored, and Safe (DREAMS) Program Reach Among Adolescent Girls and Young Women (AGYW) in Namibia: Secondary Analysis of Population and Program Data †

**DOI:** 10.3390/tropicalmed10090240

**Published:** 2025-08-27

**Authors:** Enos Moyo, Endalkachew Melese, Hadrian Mangwana, Simon Takawira, Rosalia Indongo, Bernadette Harases, Perseverance Moyo, Ntombizodwa Makurira Nyoni, Kopano Robert, Tafadzwa Dzinamarira

**Affiliations:** 1 School of Nursing & Public Health, College of Health Sciences, University of Kwa-Zulu Natal, Durban 4041, South Africa; zhua2007@gmail.com; 2 Project HOPE—The People-to-People Health Foundation, Inc., Windhoek 10005, Namibia; emelese@projecthope.org; 3 Project HOPE Namibia, Windhoek 10005, Namibia; haddi81@gmail.com (H.M.); simontakawira11@gmail.com (S.T.); rindongo@projecthope.org (R.I.); bharases@projecthope.org (B.H.); 4 Clinical Department, Medical Centre Oshakati, Oshakati 15001, Namibia; moyoperseverance@gmail.com; 5 School of Nursing, Faculty of Health, Humanities, & Social Sciences, Welwitschia University, Windhoek 10005, Namibia; rkpone2000@gmail.com; 6 School of Health Systems and Public Health, Faculty of Health Sciences, University of Pretoria, Pretoria 0001, South Africa; u19385419@up.ac.za

**Keywords:** adolescent girls and young women (AGYW), needs, Project HOPE Namibia (PHN), characterization, Determined, Resilient, Empowered, AIDS-free, Mentored, and Safe (DREAMS) project

## Abstract

Background: Namibia is experiencing a generalized HIV epidemic, with 7.5% of the population living with HIV. Adolescent girls and young women (AGYW) aged 15–24 account for 28.6% of new infections annually. Various factors increase AGYW’s vulnerability to HIV. To address this, Project HOPE Namibia (PHN)-led consortium implemented the PEPFAR/USAID-funded DREAMS project in Khomas, Oshikoto, and Zambezi regions from 2018 to 2023. This study estimated the AGYW population most in need of HIV prevention and assessed geographic and age-specific gaps to improve program effectiveness and efficiency. Methods: This secondary data analysis utilized the Namibia Population-Based HIV Impact Assessment (NamPHIA) 2017, the Namibia census, and service data from the DREAMS project, which includes entry points for recruitment, screening, and enrolment. We used Python to conduct unadjusted and adjusted Poisson regression and UpSet plots for data visualization. Results: Analysis of NamPHIA data revealed low HIV prevalence in 10–14-year-olds, with only Oshikoto showing a detectable rate of 2.76%, mostly attributed to perinatal HIV transmission. Of the 12 DREAMS eligibility criteria, three could be mapped to 10–14-year-olds, while all except sexually transmitted infections could be mapped for 15–19 and 20–24-year-olds. Nationally, 17.3% of 10–14-year-old AGYW, 48.0% of 15–19-year-olds, and 50% of 20–24-year-olds met at least one DREAMS eligibility criterion. Among 15–19-year-olds, a history of pregnancy, no/irregular condom use, and out-of-school status were positively associated with HIV status. For 20–24-year-olds, transactional sex was positively associated with HIV status. Overall, 62% of screened individuals were eligible, and 62% of eligible individuals enrolled. PHN screened 134% of the estimated 37,965 10–14-year-olds, 95% of the estimated 35,585 15–19-year-olds, and 57% of the 24,011 20–24-year-olds residing in the five districts where DREAMS was implemented. Conclusions: We recommend the refinement of the DREAMS eligibility criteria, particularly for AGYW 10–14, to better identify and engage those at risk of HIV acquisition through sexual transmission. For 15–19-year-olds, PHN efforts should interrogate geographic variability in entry points for recruitment and screening practices. PHN should enhance the recruitment and engagement of AGYW 20–24, with a particular focus on those engaged in transactional sex.

## 1. Introduction

Namibia is experiencing a generalized HIV epidemic, with 7.5% of the general population living with HIV [1]. According to the Namibia Spectrum/Naomi Model 2024, 95% of PLHIV knew their HIV status, 97% of those who knew their status were on antiretroviral treatment (ART), and 94% of all people living with HIV (PLHIV) on treatment had viral load (VL) suppression [1]. Despite these advances toward epidemic control, Namibia’s HIV prevalence remains among the highest globally [2]. Moreover, critical disparities by geography and population persist and require action to reach epidemic control. Women older than 25 years account for 33.1% of new infections, adolescent girls and young women (AGYW) 15–24 years old account for 28.6%, and men older than 25 years account for 23.8% of the new infections per year [1]. The HIV incidence among young people represented the most significant contrast. In the Namibia Population-based HIV Impact Assessment 2017 (NamPHIA 2017), the HIV incidence rate in AGYW was 1.06% compared to 0.03% in young men [3].

Many factors have been associated with increased AGYW’s vulnerability to HIV, including a history of sexually transmitted infections (STIs), alcohol use, multiple sex partners, early marriage, being out of school, no/irregular condom use, engaging in transactional sex, violence, and gender-based normative assumptions and power differentials in sexual relationships [4,5,6]. Gender-based norms reduce the ability of AGYW to successfully negotiate condom use with male partners who are exposed to HIV through their typically more extensive sexual networks [7,8]. These risks are exacerbated by men’s reluctance to access HIV services, including HIV testing (HTS), antiretroviral therapy (ART), and voluntary medical male circumcision (VMMC) [9,10]. HIV-positive males are less likely than HIV-positive females to have been diagnosed and enrolled in care and treatment, with almost 20 percent of HIV-positive males participating in NamPHIA 2017 reporting that they had never been tested [3].

**Figure 1:**
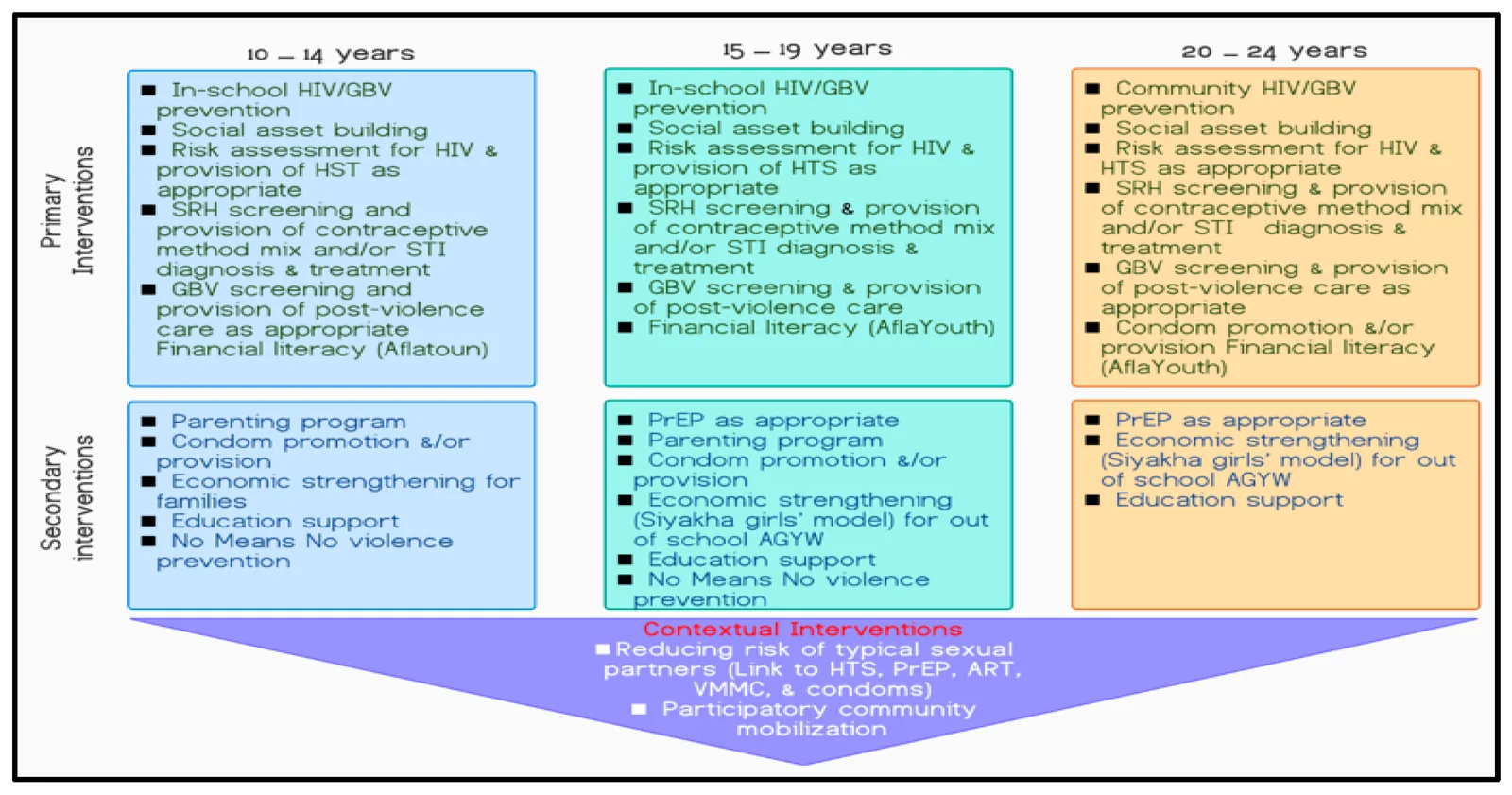
PEPFAR DREAMS Layering Table.

To address this urgent need, a consortium led by Project HOPE Namibia (PHN) implemented the PEPFAR/USAID-funded Determined, Resilient, Empowered, AIDS-free, Mentored, and Safe (DREAMS) project from 4 June 2018, to 30 June 2023, in Khomas, Oshikoto, and Zambezi regions. PHN implemented a core package of age-appropriate ‘primary’ and ‘secondary’ interventions for vulnerable AGYW 10–24 years old, outlined in the PEPFAR Namibia DREAMS layering table (See Figure 1). Moreover, interventions to strengthen families and reduce risk among sexual partners of AGYW were provided.

Since June 2020, the PHN-led DREAMS Project has utilized the PEPFAR-approved eligibility criteria summarized in Table 1. The PEPFAR-approved eligibility criteria were informed by epidemiological evidence and were updated in 2020. AGYW in their age groups meeting any one of the criteria are considered at risk of being or going ‘off-track’. Further, the prevalence of vulnerable AGYW is expected to be high in DREAMS districts, given the large number of variables and their cumulative prevalence.

Between 2018 and the first quarter (Q1) of 2023, PHN reported screening approximately 103,000 AGYW aged 10–24 for DREAMS eligibility, 57,128 (55%) of whom were eligible. The percentage of clients who were screened as eligible by age band was 62% (19,233/30,742) of 10–14-year-olds, 53% (21,770/41,235) of 15–19-year-olds, and 52% (16,125/31,019) of 20–24-year-olds. Most AGYW 10–14 years (69%) and 15–19 years (41%) reported only one eligibility criterion. PHN further noted that the more common criteria among 10–14-year-olds were the experience of physical or emotional violence in the last year (26%), any alcohol use (21%), and orphanhood (16%). Among AGYW 15–19 years, irregular condom use (23%) and orphanhood (21%) were the most prevalent criteria, and 20–24-year-olds screened positive, no/irregular condom use (61%), and multiple sex partners (10%) were the most prevalent.

The broader evidence on HIV risk suggests that DREAMS and other risk factors may vary in association with HIV geographically and temporally. Hence, a shift to prioritizing enrolment based on a risk scoring system or using a weighted criteria methodology based on the current understanding of HIV risk is an essential next step in DREAMS programming to ensure the program is identifying and enrolling the most vulnerable. We conducted this study to identify AGYW most susceptible to HIV acquisition for two reasons: (1) to appropriately allocate limited resources for the population that most needs DREAMS programming, and (2) to increase the DREAMS project’s ability to reach saturation.

In this study, we utilized NamPHIA 2017 and DREAMS Program data to risk profile AGYW participating in the DREAMS program, the coverage of the DREAMS services, and the geographical patterns of the DREAMS eligibility risk factors. The specific objectives of the study were to

- Assess HIV prevention needs of individual and conjoint DREAMS vulnerability criteria using NamPHIA data. Conjoint DREAMS vulnerability criteria in this study means two or more criteria combined.
- Determine the population size of AGYW with individual and multiple DREAMS vulnerability criteria.
- Determine the burden of infection based on individual and conjoint criteria.
- In conjunction with PHN DREAMS data from 2018, describe the level of PHN reach in DREAMS districts using baseline program-level data with population-level estimates derived from NamPHIA 2017.
- Determine the combination of criteria most predictive of HIV infection at the population level.
- Estimate DREAMS coverage on reaching the most vulnerable AGYW.
- Geo-map the DREAMS eligibility risk factors.

An abstract of this study was presented at the INTEREST conference held in Windhoek, Namibia, between 13 May and 16 May 2025 [11]. 

## 2. Methods

### 2.1. Methodological Overview

We conducted data analysis in Python version 3.12.8. As part of the systematic approach to estimate the AGYW population most in need of HIV prevention, we estimated the national, regional, and to the extent possible, district-level frequency and proportion of AGYW at HIV risk based on age-specific DREAMS 2020 criteria and other key variables of interest from prior DREAMS criteria applied during PHN programming, assessed HIV prevalence among NamPHIA AGYW in the three DREAMS age groups with singular and multiple DREAMS criteria using confirmed HIV prevalence and a modified prevalence estimate adjusting for sexual transmission as a proxy of cumulative HIV incidence and evaluated the burden of HIV infection among each AGYW age group with single and conjoint identities using confirmed HIV prevalence and a modified prevalence estimate adjusting for sexual transmission as a proxy of cumulative HIV incidence. We also described the geographic distribution of at-risk AGYW based on population size and infection burden in PHN-supported DREAMS districts, examined the association between age-specific DREAMS eligibility criteria nationally, and triangulated findings from analyses of PHN-supported DREAMS districts with other PHIA data analyses, including data quality assessments.

### 2.2. Analysis of NamPHIA Data for DREAMS Districts

#### 2.2.1. Analytic Sample

The sample of at-risk AGYW needing prevention services was defined as AGYW 10–24 years who participated in NamPHIA 2017, tested HIV negative, and resided in any of the three regions where PHN is implementing DREAMS services. The AGYW 10–24 years with HIV were included in analyses of HIV risk after removing perinatally transmitted HIV. HIV perinatal transmission refers to HIV transmission to an infant during pregnancy, delivery, or postpartum as a result of breastfeeding [12]. Behaviorally acquired HIV, on the other hand, is HIV that is acquired through risky behaviors like unprotected sex or sharing needles [13].

#### 2.2.2. Sampling

Variance estimation for variables used the Taylor method for multistage sampling weights and adjustment for nonresponse according to the detailed summary in the NamPHIA 2017 final report [3]. In brief, the first stage of sampling units was defined as the Enumeration Areas (EAs) based on the 2011 Namibia Population and Housing Census. The 2011 sampling frame comprised approximately 6200 EAs containing 497,000 households and over 2.1 million persons. A stratified probability sample of 465 EAs was selected for the 14 regions in Namibia, proportionate to the estimated number of households (occupied dwelling units). Since the number of households selected per EA was fixed at 27, unequal weighting design effects were tabulated for each stratum (region).

#### 2.2.3. Variable Definition

PHN stakeholders were consulted to identify and prioritize current and prior DREAMS eligibility criteria for inclusion, definition, and operationalization. The DREAMS indicators used in this activity and their accompanying component PHIA variables are presented in Table 2. We mapped PHIA variables to the DREAMS indicators to showcase the relationship between the two data sources. We measured HIV prevalence in two ways: (1) using the total number of HIV-positive test results (unadjusted) and (2) adjusting for the number of positive cases coming from perinatal/vertical transmission (adjusted). We defined likely cases of vertical transmission as HIV cases who (a) never had sex and (b) were never exposed to sexual violence.

#### 2.2.4. Descriptive Analysis

We generated unweighted and weighted descriptive and tabular statistics (frequencies and proportions) to estimate HIV prevalence (unadjusted, adjusted for perinatal transmission), DREAMS eligibility criteria, and the cross-tabulation of both. We plotted singular and joint prevalence (unweighted) of age-specific DREAMS eligibility criteria and their corresponding HIV prevalence at national, program, and regional levels using UpSet plots. UpSet plots are a data visualization approach to describe intersectionality with DREAMS criteria.

#### 2.2.5. Regression Analysis

We conducted unadjusted and adjusted Poisson regression to assess the association between DREAMS eligibility criteria and HIV status after adjusting for perinatal HIV in each DREAMS age band. The Poisson models were unweighted with design-based variance only. We calculated survey-adjusted F statistics to measure the statistical association between each covariate and the outcome. We included covariates in the final model if they had a statistically significant (*p* < 0.05) association with prevalent HIV infection.

#### 2.2.6. Geo-Mapping of DREAMS Eligibility Risk Factors Using NamPHIA

We examined the geographic variation in DREAMS criteria that were associated with HIV in the regression analyses. We mapped the regional prevalence of these DREAMS criteria across all of Namibia’s regions and marked the PHN-DREAMS regions from the non-DREAMS regions.

### 2.3. Analysis of DREAMS Program Data

#### 2.3.1. Project HOPE Data Management and Quality Assessment

Based on consultations with the program team, we examined the PHN program baseline data for accuracy and completeness. The data accuracy measures were based on the flow of the program such that clients at baseline should represent unique individuals with the following documented experiences: encounter location, date, and result of the meeting. Based on this program flow, we managed the data to identify the first screening encounter, the cumulative DREAMS criteria assessed, the proportion eligible, and the criteria for eligibility. We removed individuals with out-of-range values for ages < 10 and >24 years and data. Next, individuals with multiple observations were de-duplicated to identify the first screening and enrolment encounter if the individual enrolled.

**Figure 2:**
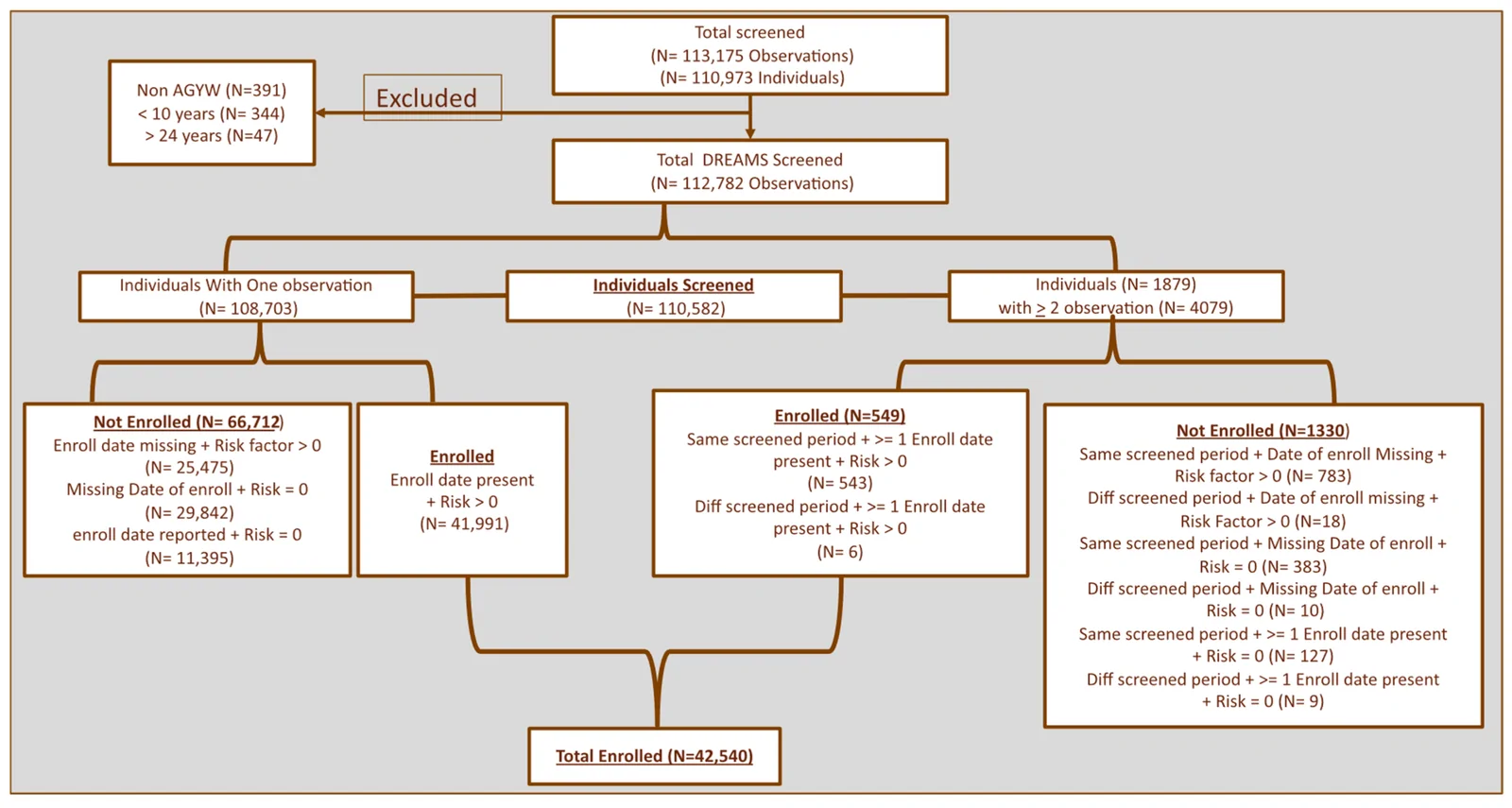
Consort diagram of preparing DREAMS analysis.

Next, we assessed the quality of enrolment status data among eligible individuals by comparing eligibility determination with the calculated number of risk factors, completion of enrolment date, and the difference between screening and enrolment dates. More details are in Figure 2. 

#### 2.3.2. Screened, Eligible, Enrolled

During the five years of PHN’s DREAMS implementation, we visualized the cascade of individuals screened, eligible, and enrolled between the ages of 10 and 24. We examined the number of screened, eligible, and enrolled individuals by eligibility period, age group, district, DREAMS entry point, and nationality.

#### 2.3.3. Reach Analysis

We defined reach as the ratio of unique individuals screened by PHN’s DREAMS program relative to the total number of individuals estimated from the Namibia census and PHIA data estimates. We developed denominators, that is, district and age-specific population size estimates, from the Namibia 2011 census extrapolations to 2017 as the population sizes. We multiplied the proportions of AGYW by the DREAMS criteria estimated from PHIA. We compared the number of unique individuals screened, “any screen,” by PHN relative to the total number of individuals residing in areas served by PHN in each DREAMS region. For Oshikoto and Zambezi, the entire population estimated for the regions was used, and the Khomas region included all districts’ census projections, excluding Windhoek East.

#### 2.3.4. Geo-Mapping of DREAMS Eligibility Risk Factors Using Project HOPE Program Data

We examined the geographic variation in PHN reach for the DREAMS criteria associated with HIV in the PHIA regression analyses. We mapped the regional prevalence of individuals estimated with these DREAMS criteria reached in the PHN-DREAMS regions.

### 2.4. Ethical Considerations

The DREAMS component of Reach PHN has been approved by the Namibian Ministry of Health and Social Services (MHSS), the Ministry of Education, Arts, and Culture (MoEAC), the Ministry of Gender Equality, Poverty Eradication and Social Welfare (MGEPESW), and the Ministry of Sport, Youth and National Service (MSYNS). All minors in the program provided assent, and their parents or caregivers granted consent. AGYW of legal age completed a consent form. Approval from an institutional review board was not necessary for the secondary data analysis due to the utilization of anonymous programmatic and publicly available NamPHIA and Namibia census data. PHN permits the utilization of its anonymized programmatic data. As per institutional policy, this secondary analysis of anonymized program and publicly available data was deemed not human subjects research and therefore, exempt from an institutional review board ethical clearance. The secondary data analysis complied with the national data-sharing agreements by ensuring that all the national data used were publicly available and the PHN data were anonymized.

## 3. Results

### 3.1. HIV Prevention Needs of Individual and Conjoint DREAMS Vulnerability Criteria Using NamPHIA 2017 Distribution of HIV

The region-specific estimates showed the HIV prevalence was highest in Zambezi (13.43%, 95% CI: 4.6–22.27), followed by Oshikoto (6.83%, 95% CI: 1.53–12.12) and Khomas (2.13%, 95% CI: =−0.26–4.53). NamPHIA results showed the weighted national and program-level (all three regions) HIV prevalence adjusted for vertical transmission in 10–14-year-olds was 1.61% (95% CI: 0.66–2.56) and 0.63% (95% CI: 0.25–1.51), respectively. Oshikoto, the only one of the three PHN DREAMS regions with detectable HIV prevalence among 10–14-year-olds, had a weighted HIV prevalence of 2.76% (95% CI: 1.03–6.54). Among 15–19-year-olds, the weighted national and program-level HIV prevalence was 4.74% (95% CI: 3.59–5.88) and 5.87% (95% CI: 3.27–8.47), respectively. Among 20–24-year-olds, the weighted national and program-level HIV prevalence were 5.99% (95% CI: 4.64–7.35) and 4.49% (95% CI: 2.34–6.64), respectively. Although the combined prevalence across the three DREAMS regions was slightly below national levels, Zambezi’s HIV prevalence (13.43%, 95% CI: 4.60–22.27) was more than twice the national prevalence reported above for AGYW. By contrast, Khomas (2.13%) and Oshikoto (6.83%) were lower than national levels.

Perinatally transmitted HIV impacted this population, accounting for all HIV cases in 10–14-year-olds and half of the infections in 15–19-year-olds, resulting in a national HIV prevalence of 4.90% and 2.68% after adjustment. In 15–19-year-olds in DREAMS regions, estimated HIV cases decreased from 21 to 10 cases after adjustment. In 20–24-year-olds, perinatal HIV had a limited influence on HIV estimates, reducing national HIV diagnoses by 4% (102 vs. 98 after adjustment).

### 3.2. Size Estimation of DREAMS Eligibility Risk Factors Among HIV-Negative AGYW, NamPHIA

#### 3.2.1. Adolescent Girls (10–14-Year-Olds)

**Figure 3:**
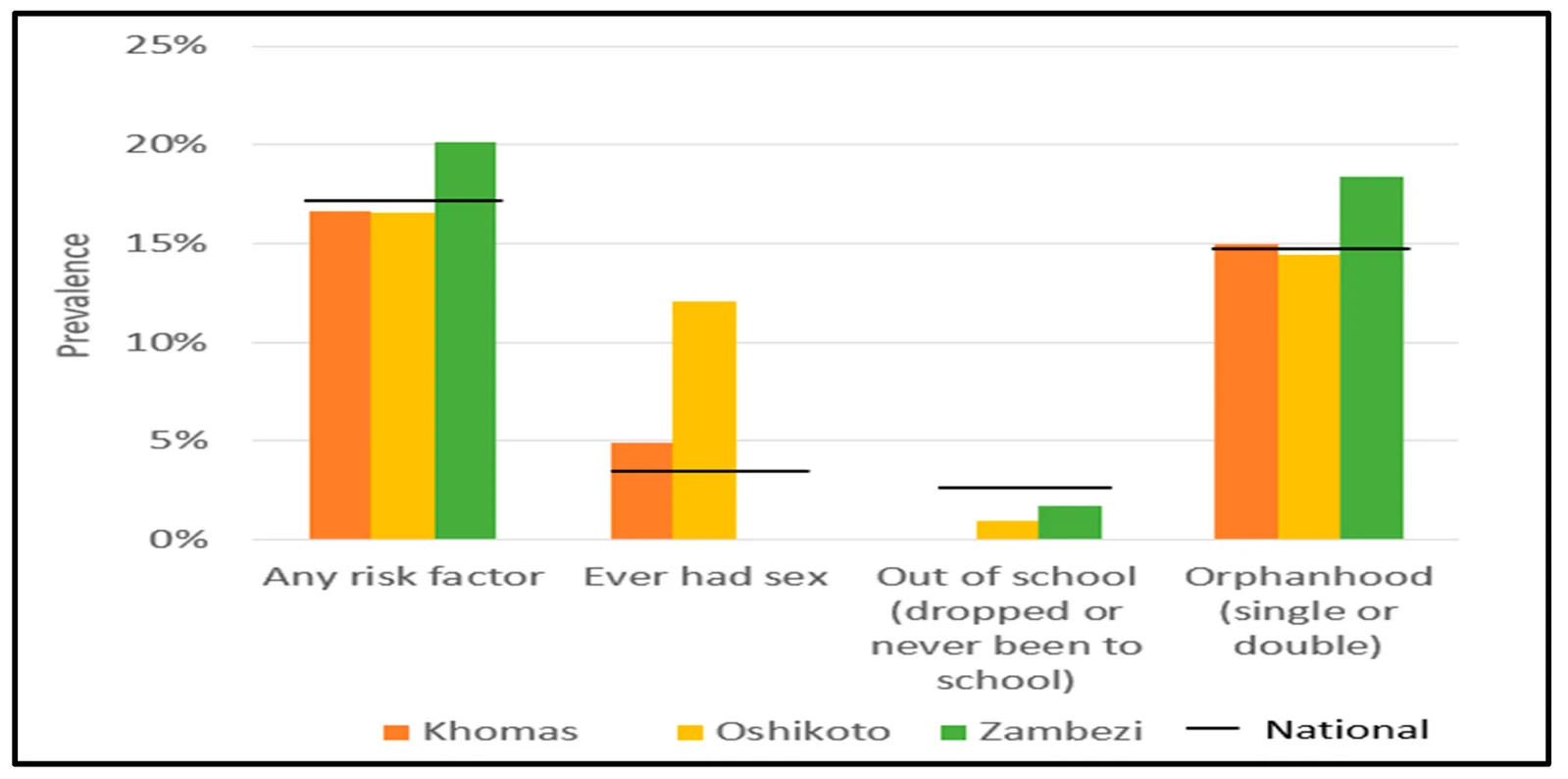
Prevalence of DREAMS Criteria in 10–14-year-olds in three DREAMS regions.

Three DREAMS eligibility criteria could be operationalized in NAMPHIA for 10–14-year-olds who had ever had sex, out-of-school, and orphanhood. Nationally, 17.30% of 10–14-year-olds had any of these three DREAMS criteria; most prevalent was orphanhood (14.66%), followed by ever having sex (3.40%) and out-of-school status (2.49%). The prevalence of DREAMS criteria in PHN-supported regions was not too different from national levels for any three criteria (17.26%) and orphanhood (15.51%). However, a history of ever having sex (5.02%) was higher than the national prevalence, and out-of-school status (0.55%) was lower. More details are in Figure 3.

#### 3.2.2. Adolescent Girls (15–19-Year-Olds)

Except for STI diagnosis, all other DREAMS criteria for 15–19 and 20–24-year-olds were mapped to the NamPHIA. Nationally, 48.01% of all 15–19-year-old AGYW had at least 1 DREAMS criterion, and DREAMS regions (47.52%) had a similar prevalence of any risk factors. The national prevalence of most other commonly reported DREAMS criteria was similar in PHN-supported DREAMS regions: no/irregular condom use (32.18% vs. 28.46%), orphanhood (23.05% vs. 24.6%), and out-of-school status (23.05% vs. 21.59%). Zambezi, the region with the most significant HIV burden in 15–19-year-olds, had a 64.84% prevalence of “any risk factor,” 33% higher than national levels. Pregnancy history (33.43%) and no/irregular condom use (32.71%), though high, were at national levels.

**Figure 4:**
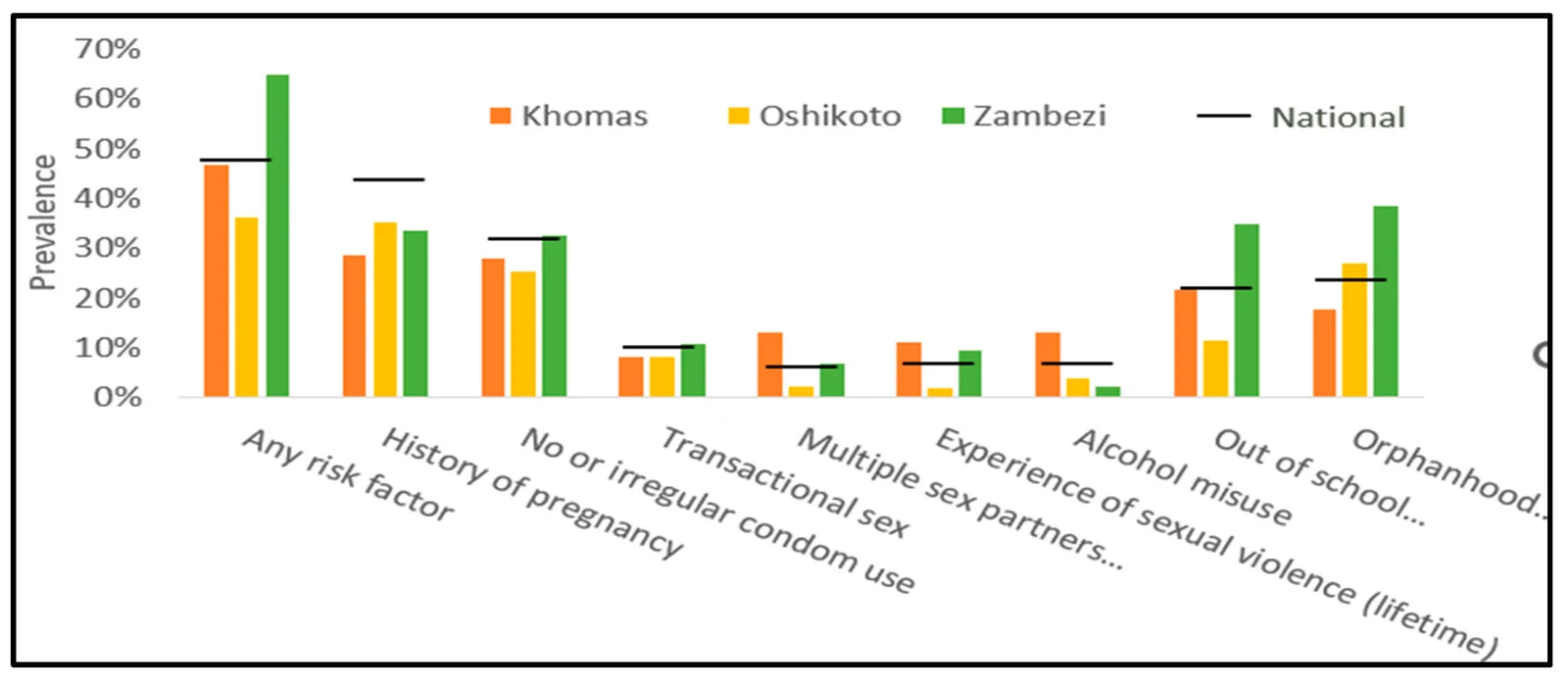
Prevalence of DREAMS Criteria in 15–19-year-olds in three DREAMS regions.

Khomas and Oshikoto, with lower HIV prevalence and levels comparable to national estimates, also had levels of DREAMS criteria that were high but comparable to national prevalence. In Khomas, the prevalence of “any risk” was 46.83%, which was mainly due to pregnancy history (28.49%), no/irregular condom use (12.67%), and out-of-school status (12.31%). Approximately 36.11% of 15–19-year-olds in Oshikoto had one or more risk factors, including pregnancy history (35.3%), no/irregular condom use (25.17%), and orphanhood (17.76%). More details are in Figure 4.

#### 3.2.3. Young Women (20–24-Year-Olds)

Nationally, 1 in 2 20–24-year-old respondents had >1 risk factor, and levels were similar in PHN-supported DREAMS regions overall (51.4%). Compared to national levels, the prevalence was identical of commonly reported risk factors like no/irregular condom use (43.99% vs. 34.55%) and alcohol misuse (16.64% vs. 19.73%) and less widely reported risk factors like transactional sex (11.37% vs. 13.66%), multiple partners (7.86% vs. 9.38%), and sexual violence survivorship (7.65% vs. 9.38%).

**Figure 5:**
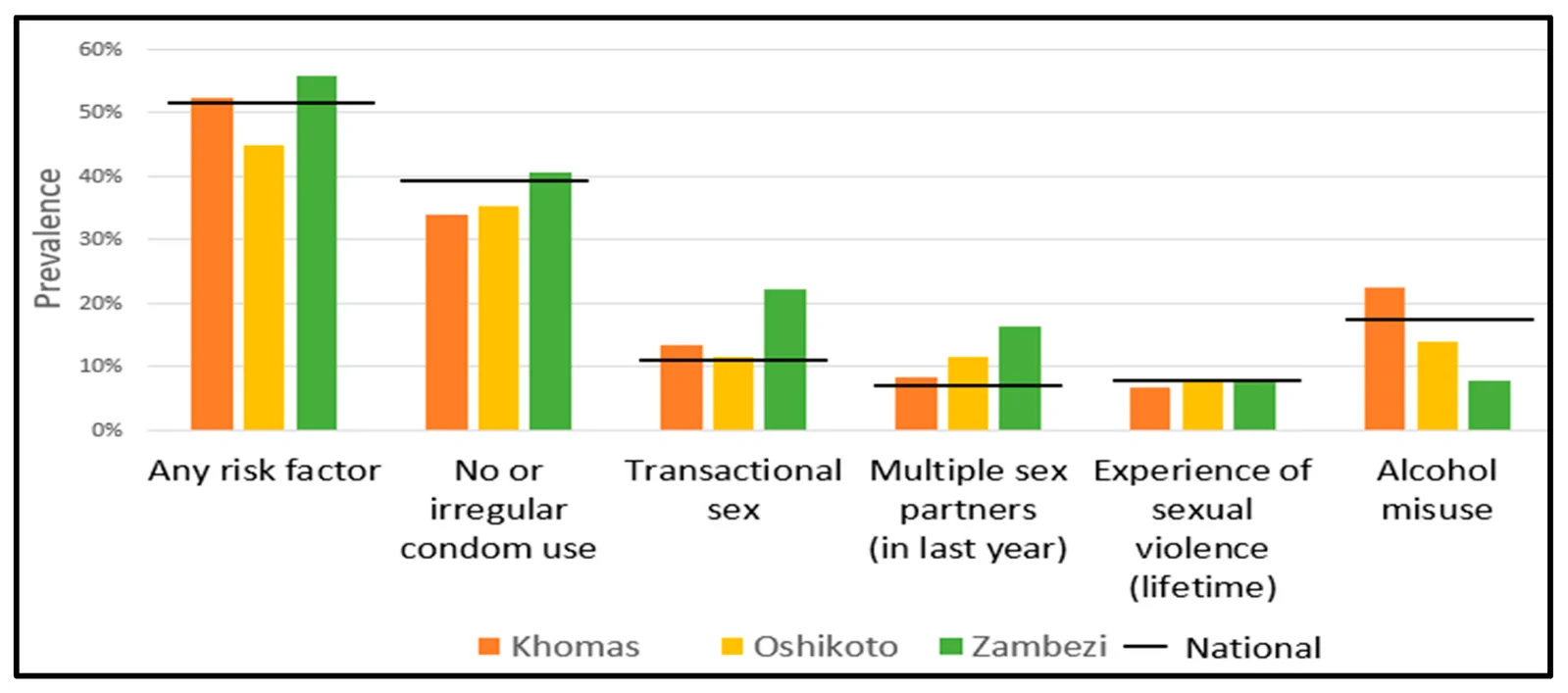
Prevalence of DREAMS Criteria in 20–24-year-olds in three DREAMS regions.

Zambezi, the region with the most significant HIV burden in 20–24-year-olds, had a 55.8% prevalence of “any risk factor,” comparable to national levels. No/irregular condom use (40.6%), transactional sex (22.06%), and multiple partnerships (16.15%) were most prevalent among 20–24-year-olds in Zambezi. In Khomas, most DREAMS criteria were at or below national levels; 52.26% had at least one risk factor, 33.74% reported no/irregular condom use, and 13.25% reported transactional sex. More details are in Figure 5.

### 3.3. Determine the Burden of Infection Based on Individual and Conjoint Criteria

In addition to examining the prevalence of HIV risk factors among HIV-negative AGYW, we examined intersectional HIV risk among all AGYW and the HIV prevalence of these individuals and overlapping risk factors. The following data are unweighted frequencies and proportions.

#### 3.3.1. Adolescent Girls (10–14-Year-Olds)

**Figure 6:**
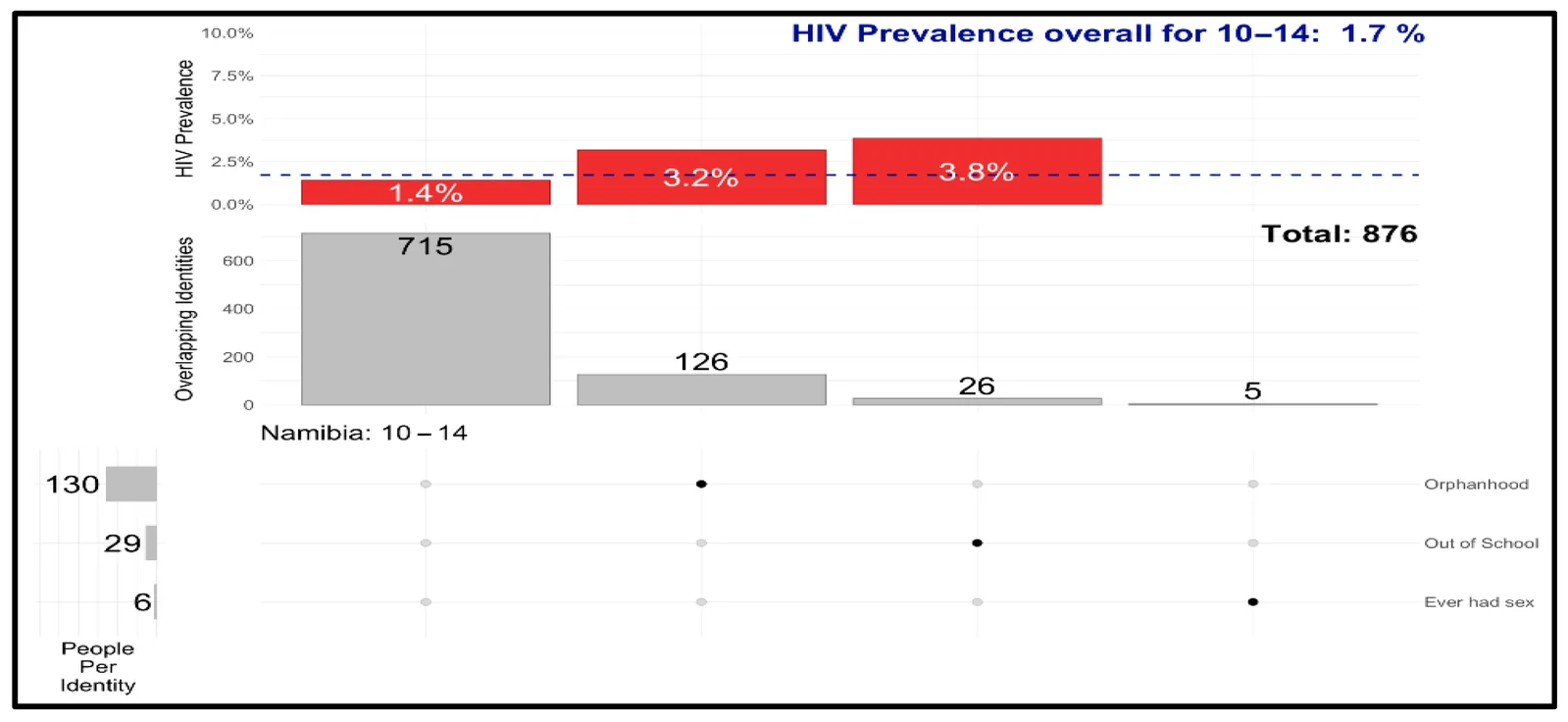
Prevalence of HIV by individual and conjoint DREAMS eligibility criteria among 10–14-year-olds in Namibia nationally.

UpSet plots of DREAMS eligibility risk criteria reported singularly or jointly among 10–14-year-olds in Namibia nationally are shown in Figure 6. The grey columns represent individuals reporting the three DREAMS criteria, the red column above represents the HIV prevalence in each group, the dashed line represents the national HIV prevalence for the age band, and the dots correspond to the singular or joint DREAMS criteria listed on the right. The HIV prevalence in the 715 10–14-year-olds who reported no DREAMS risk factors was 1.4%. Orphanhood, followed by out-of-school status, was the most reported risk factor, with HIV prevalence exceeding the national average. The joint combination of risk factors was reported nationally at very low frequencies in this age band. Yet HIV prevalence was highest among those out of school (3.8%).

**Figure 7:**
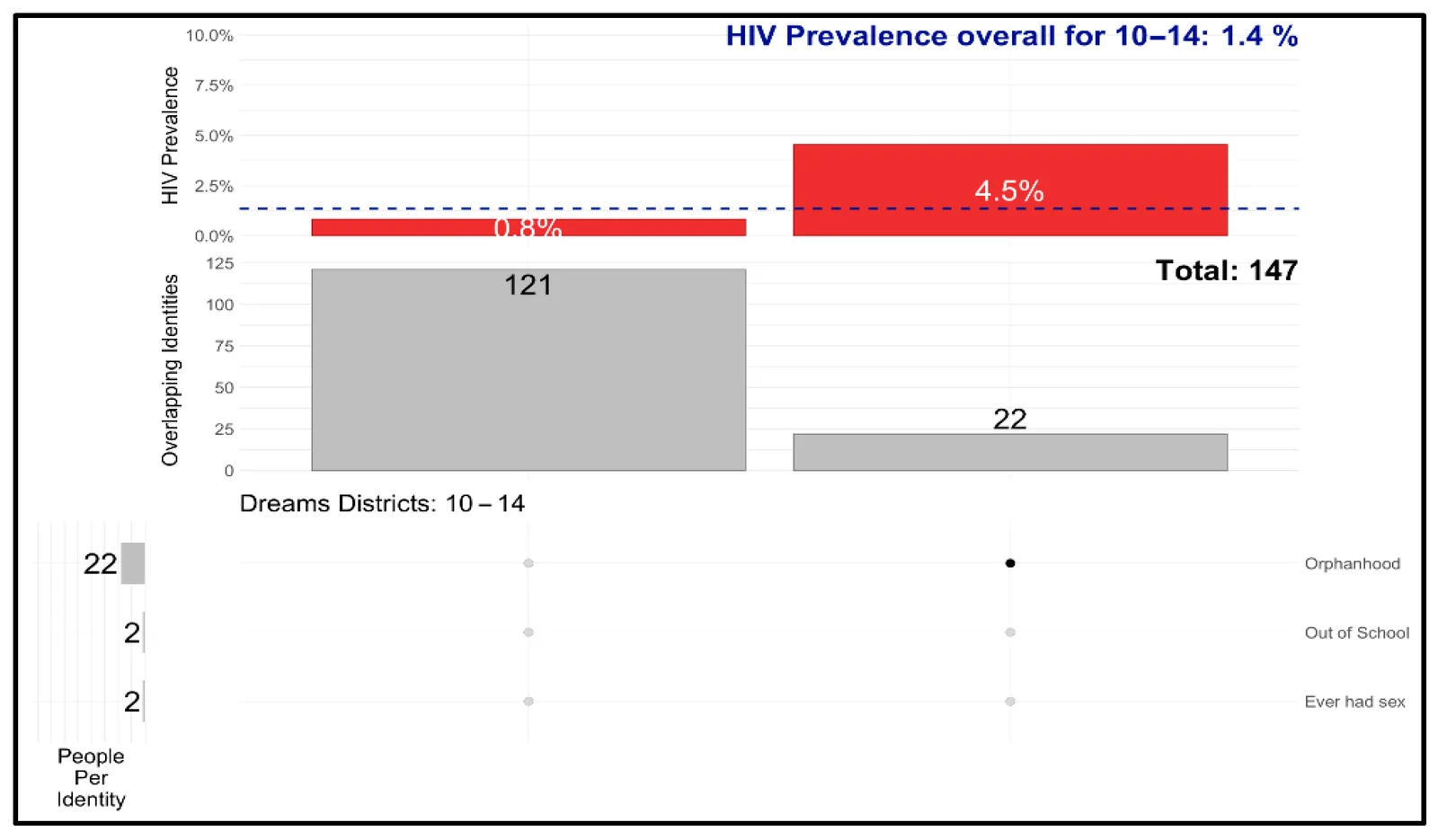
Prevalence of HIV by individual and conjoint DREAMS eligibility criteria among 10–14-year-olds in all three PHN-supported DREAMS regions.

UpSet plots among 10–14-year-olds in PHN-DREAMS regions are shown in Figure 7. Like national trends, orphanhood was the most common risk factor, with an HIV prevalence of 4.5%, exceeding the 1.4% national HIV prevalence.

**Figure 8:**
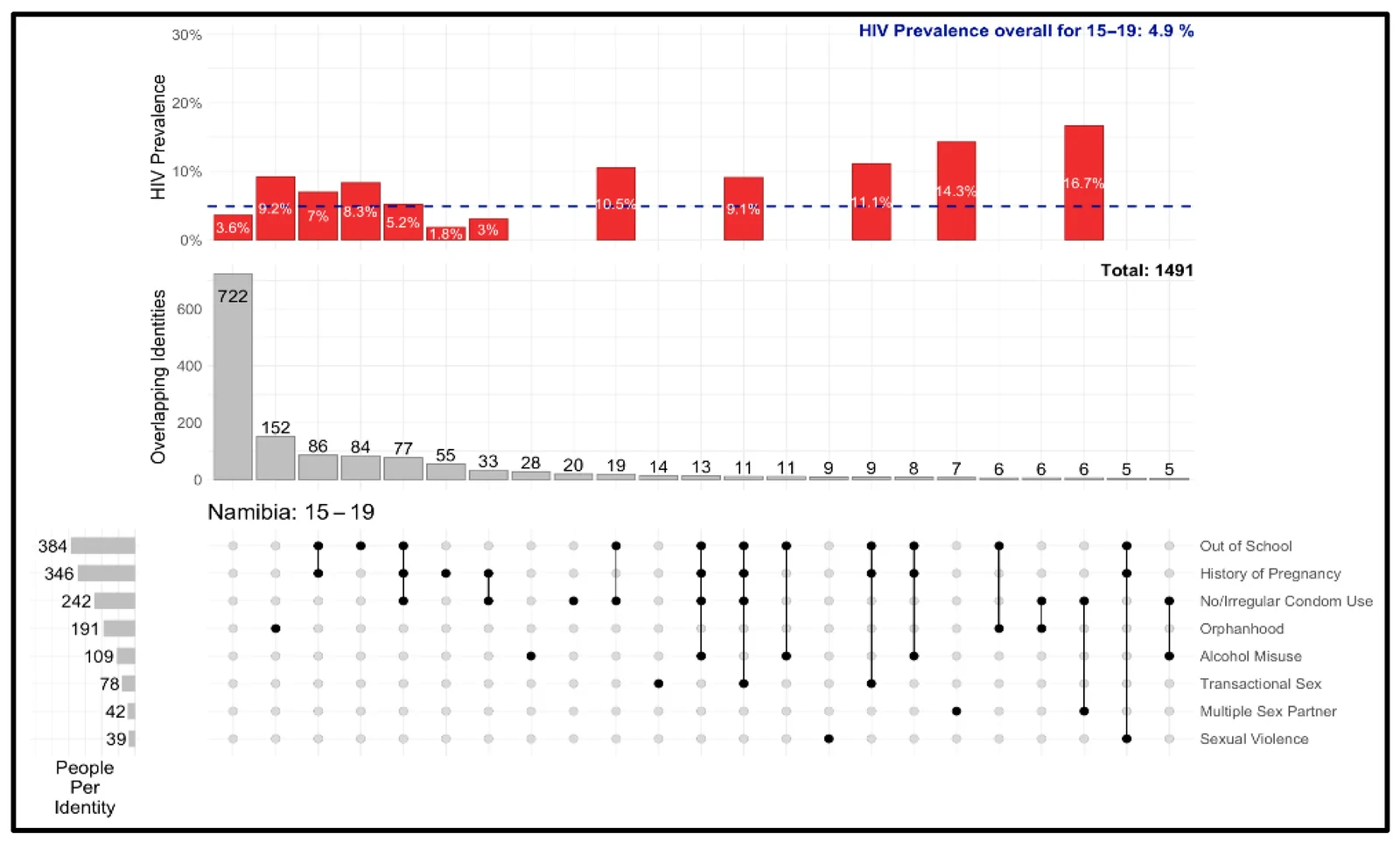
Prevalence of HIV by individual and conjoint DREAMS eligibility criteria among 15–19-year-olds in Namibia nationally.

Singular and joint DREAMS eligibility criteria reported among 15–19-year-olds nationally, with frequencies of 5 or higher, are shown in Figure 8. Individuals reporting no DREAMS criteria (N = 722) had HIV prevalence slightly below the national average. Individuals reporting orphanhood as the most prevalent criterion had an HIV prevalence of 9.2%. The next more prevalent group was those reporting the combination of Out-of-school status and pregnancy history, with an HIV prevalence of 7%. The following most prevalent combination included no/irregular condom use, with the combined school and pregnancy status having an HIV prevalence of 4.9%. The HIV prevalence was elevated above the national level for the individual and combined DREAMS criteria. Among respondents reporting a single risk factor, AGYW who reported multiple sexual partners had the highest HIV prevalence of 14.3%, representing 42 respondents. Other combinations had HIV prevalence exceeding the national average, but these combinations represented few respondents.

**Figure 9:**
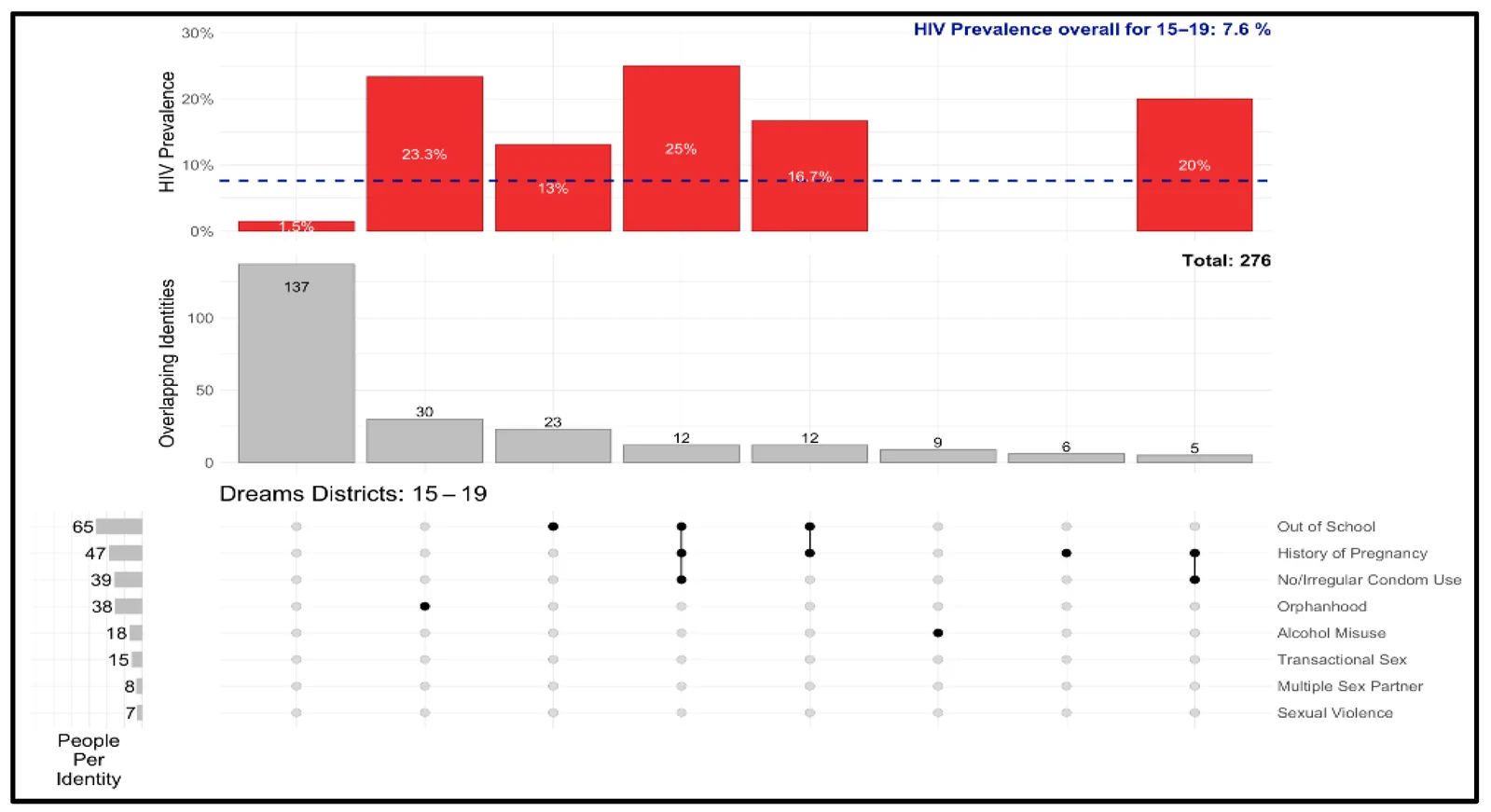
Prevalence of HIV by individual and conjoint DREAMS eligibility criteria among 15–19-year-olds in all three PHN-supported DREAMS.

UpSet plots of DREAMS criteria among 15–19-year-olds in PHN-DREAMS regions are shown in Figure 9. We present combinations of DREAMS criteria with a frequency of five or more. Like national patterns, orphanhood alone was the most prevalent risk factor (79% not grouped), and the HIV prevalence was 23.1%. Alcohol misuse alone was the second most reported DREAMS criterion (50% not grouped), and the HIV prevalence was 0.0%. Out-of-school status was the third-most isolated factor (35% not grouped), with an HIV prevalence almost twice the overall prevalence of 7.6%. Out-of-school status, pregnancy history, and no/irregular condom use and out-of-school status and pregnancy history were the most common groupings, each having a respective HIV prevalence that was thrice and twice as high as the overall prevalence.

#### 3.3.2. Young Women (20–24-Year-Olds)

**Figure 10:**
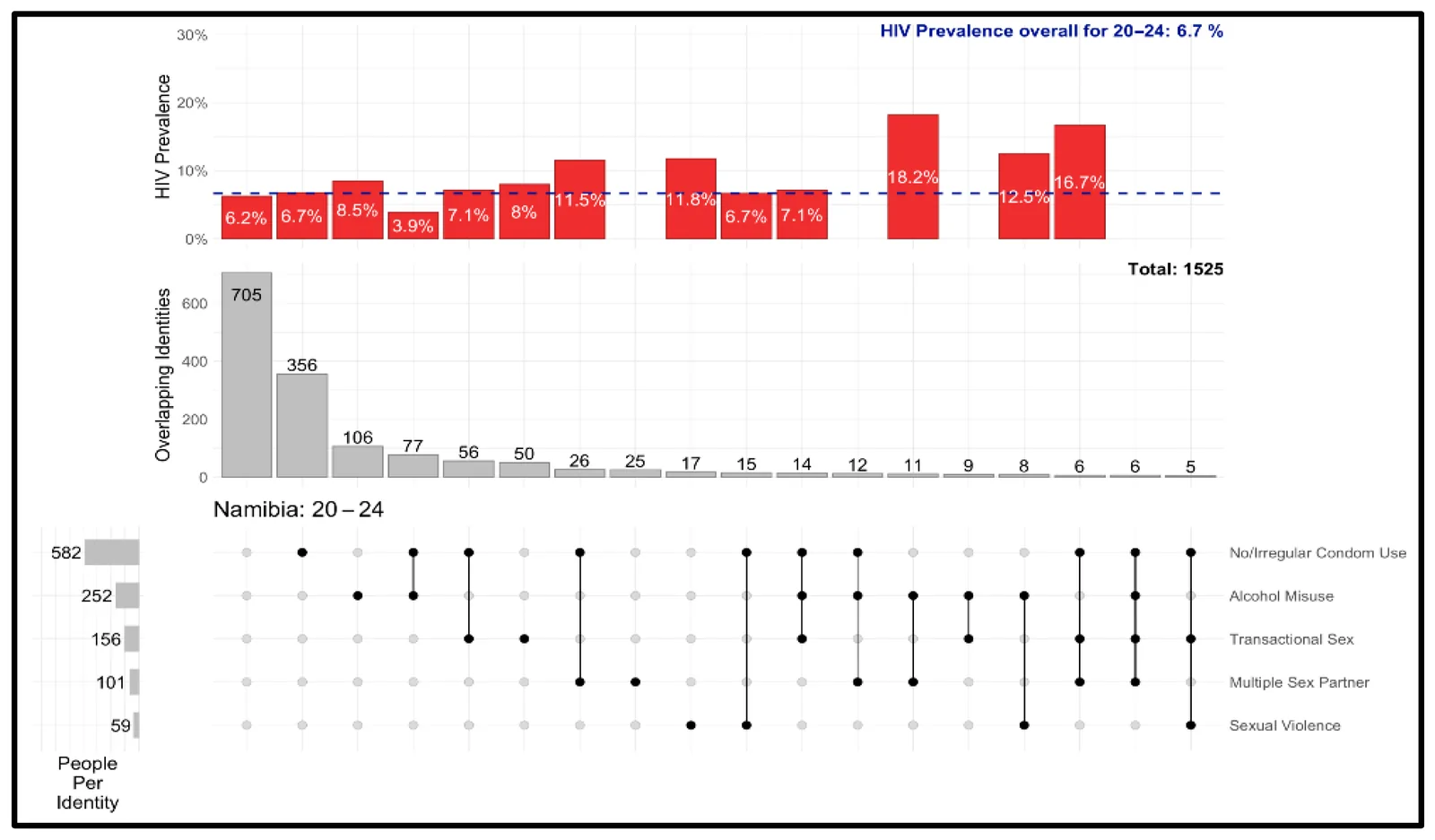
Prevalence of HIV by individual and conjoint DREAMS eligibility criteria among 20–24-year-olds in Namibia nationally.

Singular and joint DREAMS eligibility risk criteria among 20–24-year-olds nationally are presented in Figure 10. No/irregular condom use was the most common criterion, followed by alcohol misuse and transactional sex. No/irregular condom use was the most reported alone (61% ungrouped), with a similar HIV prevalence as the overall prevalence: 6.7%. Alcohol misuse (42% ungrouped) and transactional sex (32% ungrouped) were the second and third-most isolated factors, and each had an HIV prevalence that exceeded the overall average. No/irregular condom use and alcohol misuse were the most common pairing, but had an HIV prevalence under the overall average; no/irregular condom use and transactional sex were the second-most common pairing, with an HIV prevalence slightly higher than the overall average.

**Figure 11:**
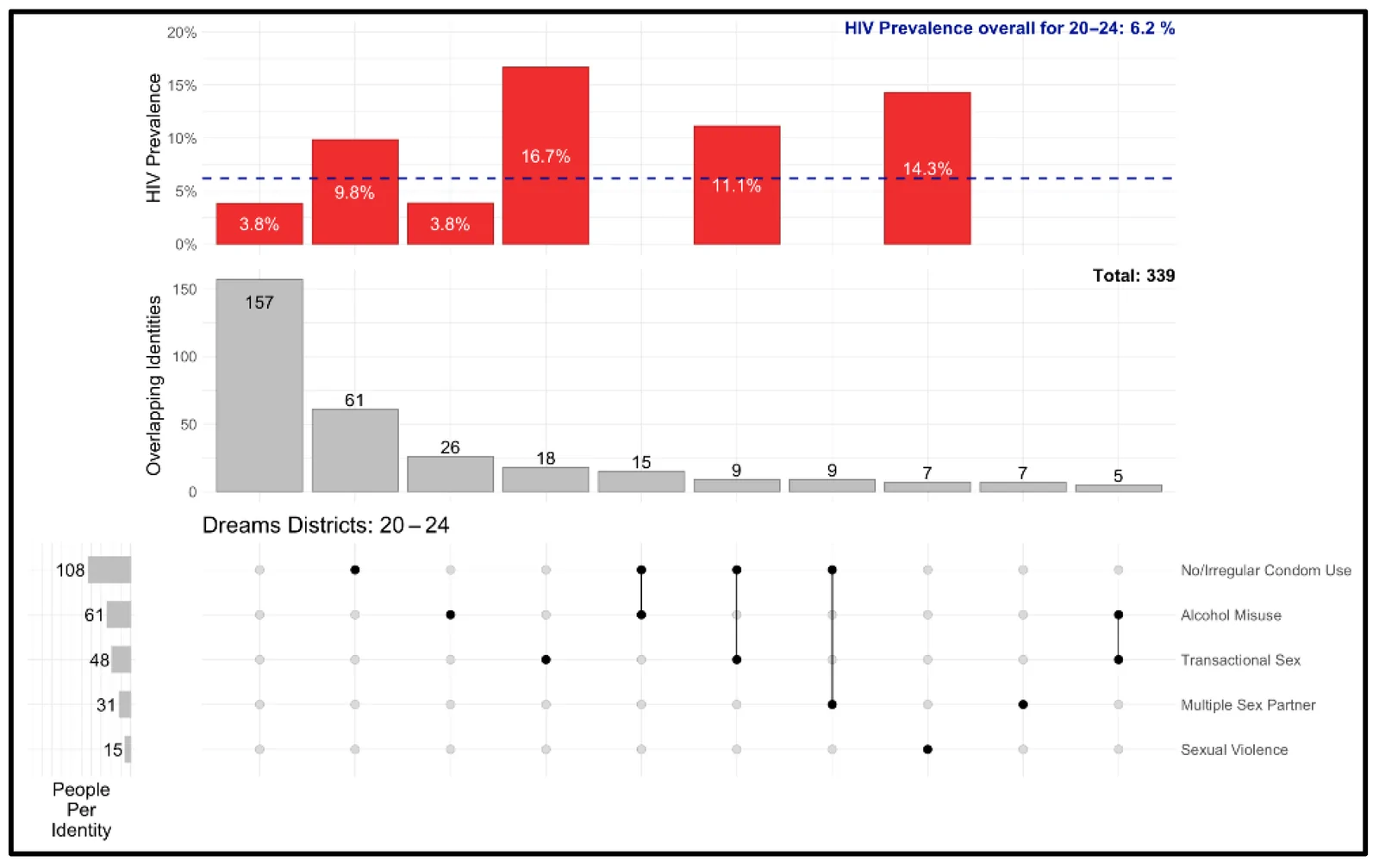
Prevalence of HIV by individual and conjoint DREAMS eligibility criteria among 20–24-year-olds in all three PHN-supported DREAMS regions.

UpSet plots of DREAMS eligibility risk criteria among 20–24-year-olds in DREAMS regions are shown in Figure 11. No/irregular condom use, alcohol misuse, and transactional sex were the most common risk factors. No/irregular condom use (56% ungrouped), sexual violence survivorship (47%), and alcohol misuse (43% ungrouped) were the most isolated factors, and all three had an HIV prevalence above the overall average of 6.7%. Of the three most common co-occurrences (no/irregular condom use + alcohol misuse, no/irregular condom use + transactional sex, no/irregular condom use + multiple sexual partners), only no/irregular condom use + transactional sex had an HIV prevalence that was almost two times the overall average.

### 3.4. Determining the Criteria Combination Predictive of HIV Infection at the Population Level

We conducted Poisson regression analyses to assess which DREAMS eligibility criteria were associated with HIV status adjusted for perinatal HIV. Among 15–19-year-olds, a history of pregnancy (RR: 5.98 [1.81–19.82]), no/irregular condom use (RR: 4.46 [1.11–18.00]), and out-of-school status (RR: 6.14 [1.33–28.29]) were positively associated with HIV status. No factors were associated with HIV status in the adjusted model. For 20–24-year-olds, transactional sex (RR: 4.69 [1.93–11.39]) was positively associated with HIV status. Since only one factor was associated with HIV status in this age group, an adjusted model was not necessary.

### 3.5. Exploration of Geographical Variation in DREAMS Eligibility Risk Factors That Were Positively Associated with HIV Status in Poisson Regression Analyses

The geographic distribution of DREAMS criteria associated with HIV in NamPHIA 2017 using Poisson regression analysis is discussed in the sections that follow.

#### 3.5.1. Adolescent Girls (15–19-Year-Olds)

**Figure 12:**
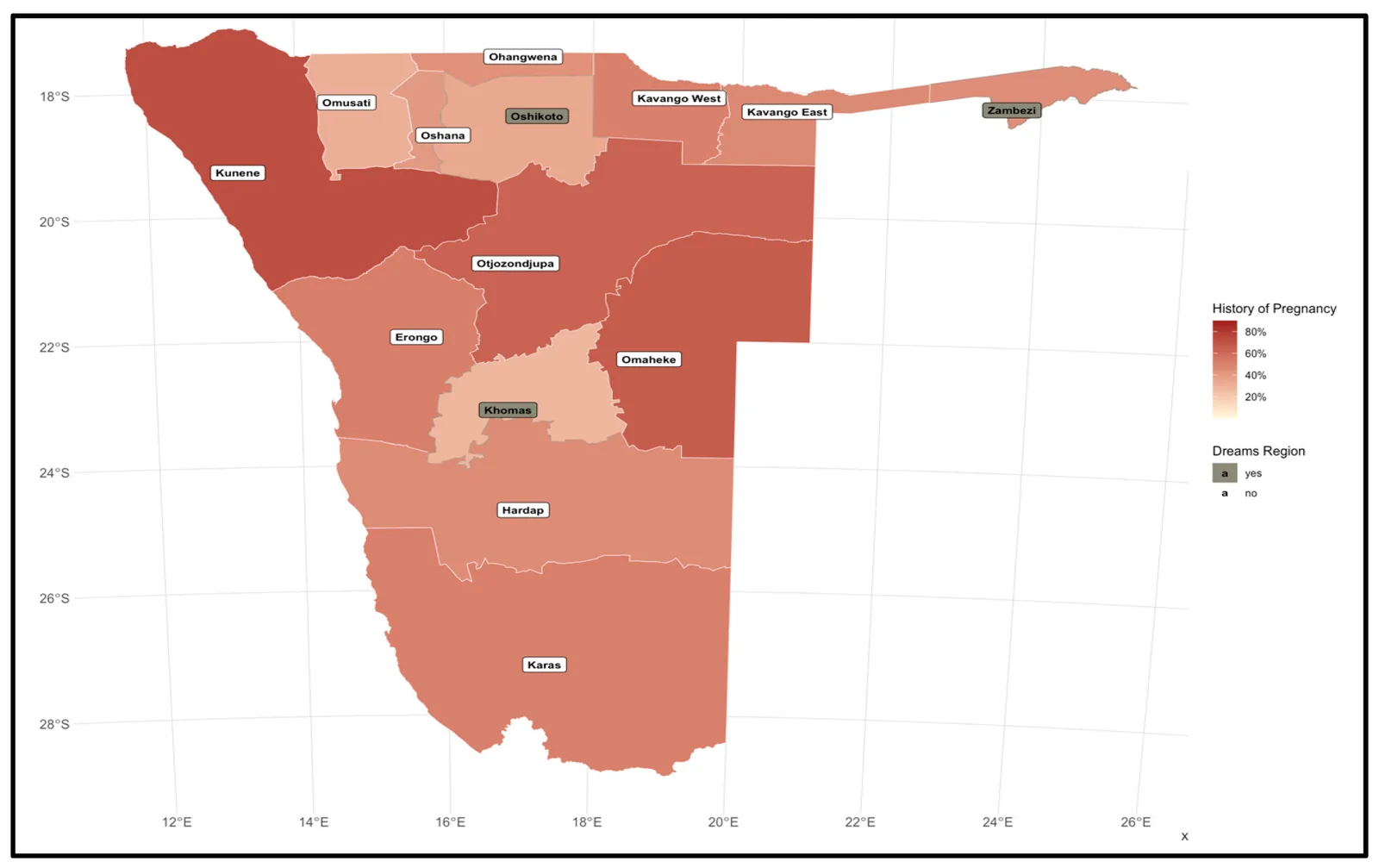
Pregnancy history among 15–19-year-olds who reported ever having sex.

The geographic distribution of pregnancy history among ever-sex 15–19-year-olds is shown in Figure 12. Nationally, pregnancy history ranged from 20 to 80% of 15–19-year-olds. The geographic (regional) variation in the pregnancy history reported by 15–19-year-olds did not highlight PHN-DREAMS regions as different from others. Interestingly, DREAMS were on the lower end of the distribution of pregnancy history. Within the DREAMS regions, pregnancy history in this group was highest in Zambezi, followed by Oshikoto and Khomas.

The geographic variation in reported no/irregular condom use among 15–19-year-olds who reported having had sex was between 20% and 80%. Like the pregnancy experience, the geographic variation did not distinguish the DREAMS regions from others. Indeed, the PHN regions were on the lower end of the geo-distribution of reported no/irregular condom use. Among the DREAMS regions, Zambezi had the highest no/irregular condom use, while Oshikoto and Khomas had relatively similar distributions. The prevalence of out-of-school girls across regions was notably high. The DREAMS regions did not have the highest concentration of out-of-school 15–19-year-olds. Among the PHN-DREAMS regions, Zambezi had the highest concentration of out-of-school 15–19-year-olds, followed by Khomas and Oshikoto.

#### 3.5.2. Young Women (20–24-Year-Olds)

**Figure 13:**
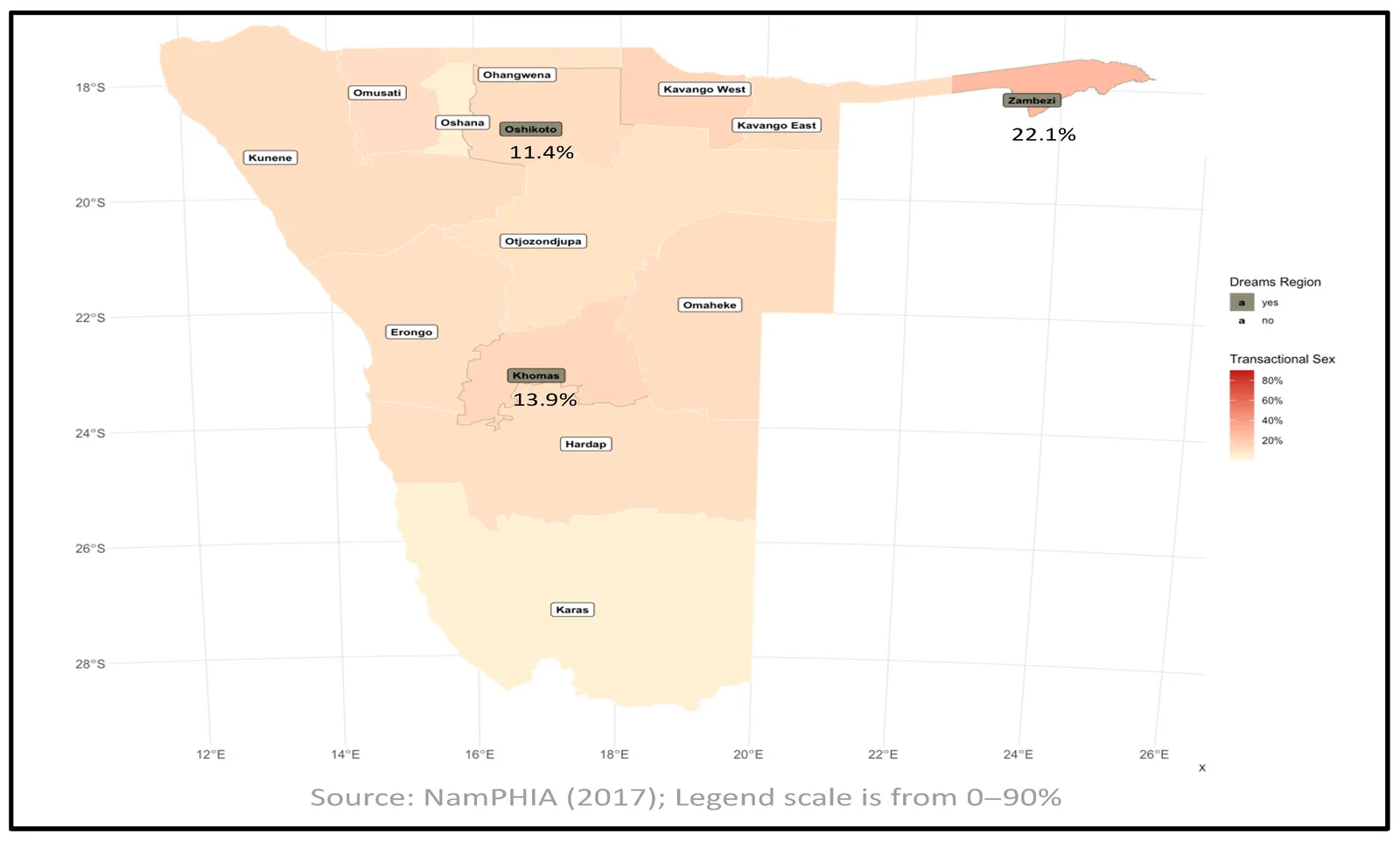
Transactional sex among 20–24-year-olds who reported ever having sex.

The geographic distribution of transactional sex among 20–24-year-olds who have ever had sex is shown in Figure 13. Although transactional sex was relatively rare across regions, the DREAMS regions contained some of the highest proportions of reported transactional sex, with Zambezi and Khomas having the most elevated and second-highest ratios in the country.

### 3.6. Determining the Population Size of AGYW with Individual and Multiple DREAMS Vulnerability Criteria

Establishing the Analytic Sample from Program Data to Estimate Reach

Figure 2 shows 110,973 individuals with 113,175 observations. After excluding individuals with age (i.e., <10 years or aged >24 years), 110,582 individuals remained in the program dataset, 108,703 of whom had been screened once and 1879 had been screened twice or more times during the five years of PHN’s implementation. After de-duplicating records, 68,816 reported at least one DREAMS eligibility criterion at any time or eligibility period. Among the eligible 68,816, a total of 42,540 enrolled in DREAMS.

**Figure 14:**
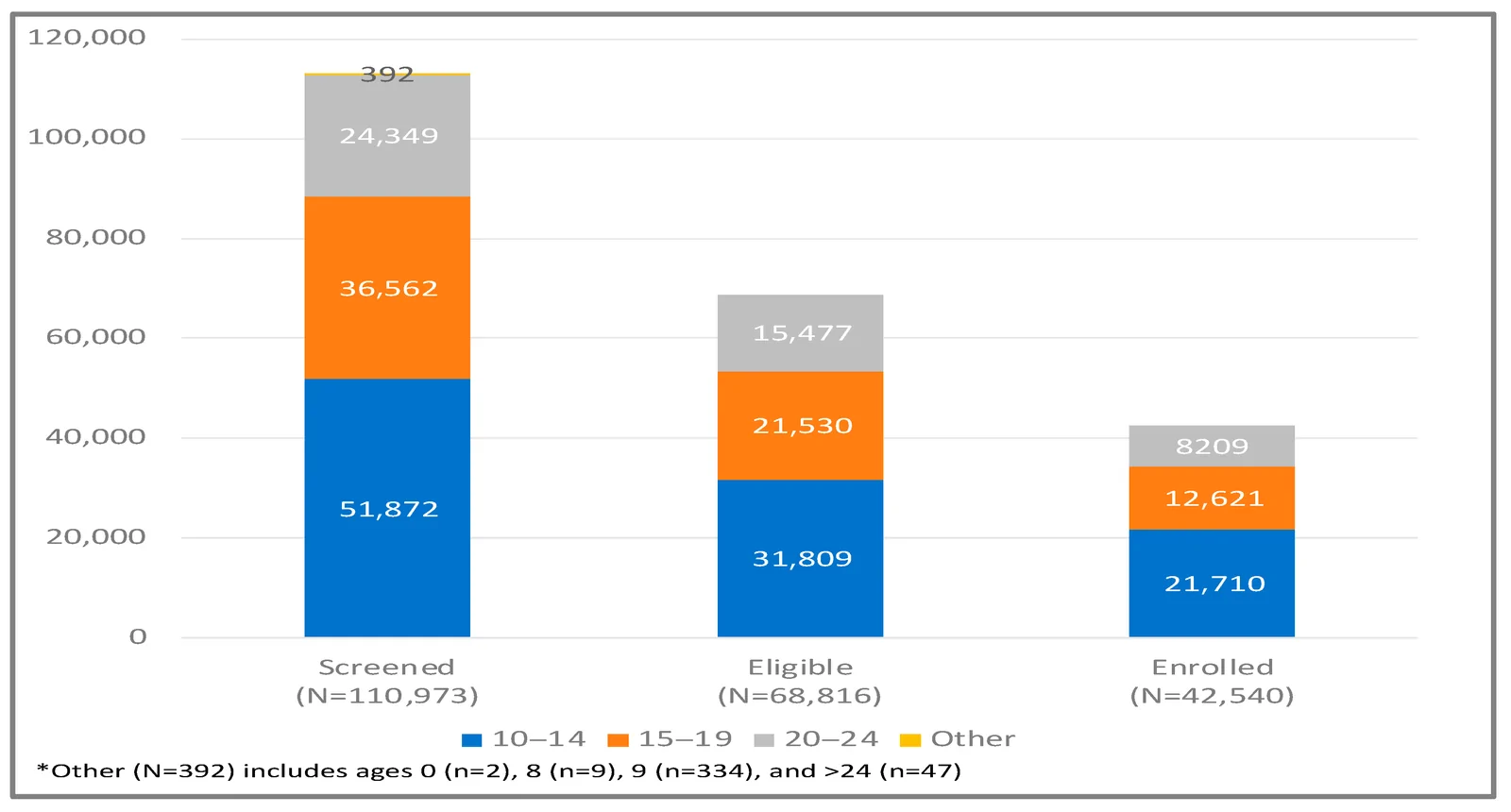
Screening, eligibility, and enrolment cascade of PHN clients screened, 2018–2023.

Figure 14 displays the program screening-eligible-enrolment cascade for the cumulative five years. Overall, 62% of screened individuals were eligible, and 62% of eligible individuals enrolled. Of all people screened, 38% ended up enrolling.

Most screenings occurred in the 1 December 2020-Present (46.5%) eligibility period, followed by 4 June 2018–29 February 2020 (41.2%). Screenings were lowest during the 1 March 2020–30 November 2020, period, some of which may be attributed to the emergence of the COVID-19 pandemic. The screening efficiency improved in each eligibility period: 25%, 51%, and 71%, respectively. The most screened age group was 10–14-year-olds (46%), followed by 15–19-year-olds (32%) and 20–24-year-olds (22%). However, the proportion identified as eligible was similar across age bands (61–64%). Although 20–24-year-olds were the least screened, they had the highest proportion of eligible individuals (65% screened were eligible), then 10–14-year-olds (62% screened were eligible) and 15–19-year-olds (61% screened were eligible). The proportion of eligible individuals screened was similar across districts. The ratio of eligible individuals screened by DREAMS entry points ranged from 67% in schools, 81% in health facilities, and 93% at locations not clearly described. The age band of 20–24-year-olds had the lowest proportion of enrolments (53% of eligible individuals enrolled) compared to 59% of eligible 15–19-year-olds enrolled and 60% of eligible 10–14-year-olds enrolled. Most screenings occurred in Windhoek (39%), Katima (21%), and Onandjokwe (17%), and most enrolments happened in these districts, too (37%, 23%, and 20%, respectively). Schools and community outreach were the first and second most common entry points for reaching potential individuals for screening and enrolment. The preponderance of individuals reached via screening and enrolment were born in Namibia.

### 3.7. Program Reach

The catchment for PHN represented full regional coverage in Oshikoto and Zambezi, but not in Khomas. PHN did not operate in Windhoek East, so the population size estimation, the denominators in the reach estimate for each age group, was adjusted by subtracting the 2017 census estimate for Windhoek East from the total 2017 census size estimation for each criterion. We multiplied the prevalence of each risk factor estimated in PHIA by the 2017 census size estimate for each region. The regional denominators were calculated by multiplying the 2011 census population projected to 2017 by the NamPHIA criterion prevalence. For Khomas, Windhoek East was excluded from the denominator.

#### 3.7.1. Adolescent Girls 10–14-Year-Olds

For 10–14-year-olds, Khomas’ regional population was 18,703, of which 904 resided in Windhoek East. Thus, we corrected the denominators using a proportion of 95.2% (17,799/18,703).

PHN screened 134% of the estimated 37,965 10–14-year-olds residing in the three regions and five districts where DREAMS was implemented. PHN screened 2.3 times the 6040 10–14-year-olds with any of the three DREAMS criteria estimated based on the NAMPHIA prevalence. Screening reach levels were estimated to be 19.10, 2.09, and 1.15-fold above the estimated number of 10–14-year-olds who had ever had sex, were out-of-school, or were orphaned. Region-specific reach was estimated at 97% in Khomas, 168% in Oshikoto, and 167% in Zambezi. In Khomas, we estimate that 58% of individuals identifying as orphans were reached via screening throughout the program, and most other characteristics approached or exceeded population size estimates. In the Oshikoto and Zambezi regions, screening reach exceeded the population sizes.

#### 3.7.2. Adolescent Girls (15–19-Year-Olds)

Among 15–19-year-olds, the regional population of Khomas was 20,708, with 953 living in Windhoek East. We multiplied their denominators by 95.4% (19,755/20,708).

PHN screened 95% of the estimated 35,585 15–19-year-olds residing in the three regions and five districts where DREAMS was implemented. PHN screened 94% of the 16,465 15–19-year-olds with at least one DREAMS criterion estimated based on the NAMPHIA prevalence. Screening reach levels were estimated to be 47% out of school, 85% of alcohol misuse, 93% of the history of pregnancy, and 93% of transactional sex. For the following DREAMS criteria, screening exceeded the estimated population: 117% experienced violence, 139% of orphans, 146% of multiple sex partners, and 205% of clients with no/irregular condom use. In Khomas, we estimated 67% of AGYW 15–19 years were screened, and varying levels of screening coverage. The lowest level of reach was observed among AGYW reporting out-of-school (46%) and misuse of alcohol (47%). The proportion of AGYW 15–19 who were reached with screening in Oshikoto and Zambezi exceeded the population size estimation by 129% and 124%, respectively. In Oshikoto, the screening reach exceeded the size estimation for all DREAMS criteria. In Zambezi, screening reach exceeded the size estimation for most DREAMS criteria except out-of-school (23%) and sexual violence (55%).

#### 3.7.3. Young Women (20–24-Year-Olds)

Overall, 29,743 20–24-year-olds lived in Khomas. Among them, 1208 resided in Windhoek East. The correction proportion was, thus, 95.9% (28,534/29,742)

PHN screened 57% of the 24,011 20–24-year-olds residing in the three regions and five districts where DREAMS was implemented. PHN screened 62% of the 13,944 20–24-year-olds with at least one DREAMS criterion estimated based on the NamPHIA prevalence. Screening reach coverage was estimated to be as high as 100% of AGYW who had no/irregular condom users and as low as 31% and 22% of AGYW engaged in transactional sex and alcohol misuse, respectively. In Khomas, we estimated that 43% of AGYW aged 20–24 years were screened and had varying levels of screening coverage. The lowest level of screening reach was observed among AGYW reporting misuse of alcohol (14%), transactional sex (21%), and as high as 74% among no/irregular condom use. The proportion of AGYW 20–24 who were reached with screening in Oshikoto and Zambezi was 76% and 106%, respectively. In Oshikoto, the screening reach exceeded the size estimation for no/irregular condom use, and all other DREAMS criteria had a screening reach of 41% (alcohol misuse) to 65% for multiple sex partners. In Zambezi, screening reach exceeded the estimated population size with the following DREAMS criteria: no/irregular condom use (219%) and alcohol misuse (164%). The lowest estimated screening reach was 33% of those experiencing sexual violence, followed by 67% of transactional sex.

### 3.8. Geo-Mapping of DREAMS Eligibility Risk Factors Using Program Data

The geographic variation in PHN reach for the DREAMS criteria associated with HIV was analyzed using the PHIA regression analyses.

#### 3.8.1. Adolescent Girls (15–19-Year-Olds)

Khomas had the lowest reach of ever-pregnant 15–19-year-olds of the three regions, with 69%. In contrast, reach targets were exceeded in Zambezi (110%) and Oshikoto (122%). More details are in Supplementary Figure S1.

Reach targets of no/irregular condom users were exceeded in all three regions: Khomas (162%), Zambezi (276%), and Oshikoto (228%). Of the three factors examined in this section, reach was lowest for out-of-school girls. The only region to reach the target population was Oshikoto (105%), while Khomas (46%) and Zambezi (23%) were both <50%.

#### 3.8.2. Young Women (20–24-Year-Olds)

While not all 20–24-year-olds reporting transactional sex were reached, PHN reach in Zambezi was 67%, 46% in Oshikoto, and 21% in Khomas. More details are in Supplementary Figure S2.

## 4. Discussion

Analysis of the NamPHIA 2017 highlighted that few HIV infections were detected among 10–14-year-olds, and of those detected, almost all could be accounted for through perinatal transmission. These results are similar to those reported by a South African study, which revealed that HIV prevalence was lowest among 10–14-year-olds [14]. The finding that almost all HIV infections could be accounted for through perinatal transmission was expected since most of the children in this age group may not be sexually active. The most plausible explanation for their HIV status could be the perinatal transmission, where the mothers were not enrolled in prevention of mother-to-child transmission programs [15].

The prevalence of HIV in 15–19-year-olds was about 5% overall, and regional prevalence estimates ranged from 3.2% in Khomas, 7.8% in Oshikoto, and 14.8% in Zambezi. Perinatally acquired HIV accounted for nearly 50% of observed HIV infection in 15–19-year-olds. However, the proportion varied by region, with Zambezi accounting for the highest level and proportion of behaviorally acquired HIV. The relatively high prevalence of HIV in Zambezi could be attributed to orphanhood (38.52%) and out-of-school status (34.81%), which exceeded national levels. Among AGYW aged 20–24, the HIV prevalence was 6.7% overall, with the prevalence in Khomas and Oshikoto remaining around 3.1% and 23.5% in Zambezi. About 4% of HIV infections were estimated to be perinatally acquired. Still, the assumptions informing the algorithm for vertical transmission are less reliable at 20–24-year-olds, where assumptions were more challenging to apply and validate in this age band. A Kenyan study also revealed that perinatal transmission decreased with age among adolescents and young people [16]. Therefore, secondary prevention among AGYW aged 15–19 should be a critical component of HIV prevention programs [17,18].

The number of HIV infections detected was few, so we examined the association between DREAMS criteria and behaviorally acquired HIV in Namibia using Poisson regression. Examining the association between DREAMS criteria and behaviorally acquired HIV was important because DREAMS indicators like sexual violence, transactional sex, no/irregular condom use, and multiple sexual partners are risk factors for sexually transmitted infections (STIs), which increase the risk of HIV infection [19]. The integration of STI screening into the DREAMS interventions was informed by the literature on the association between STIs and HIV. We could not conduct robust statistical analyses of the age-specific associations at the national, program, or regional levels. Regression models could not be developed for 10–14-year-olds. Among 15–19-year-olds, we observed in unadjusted Poisson regression analyses that a history of pregnancy, no/irregular condom use, and out-of-school status were associated with a 5.98, 4.46, and 6.14 increased risk of behaviorally acquired HIV, respectively. Although the confidence intervals did not include the null for many estimates, the 95% confidence intervals were extensive, and estimates were minimal, suggesting statistically unstable statistics. After adjustment, no variable was associated with HIV. The lack of statistical significance of the risk factors after adjustment may point toward limited statistical power of the study or a need to refine the eligibility criteria for the DREAMS program. Among 20–24-year-olds, transactional sex was associated with a 4.69 times increased risk of behaviorally acquired HIV. This was the only DREAMS criterion related to HIV in this age band. It is noteworthy that all other DREAMS criteria, except alcohol misuse, had relative risk estimates showing an increased HIV risk, but associations did not meet statistical significance in 20–24-year-olds. The lack of statistical significance of the other risk factors in the 20–24-year-olds may be attributed to social desirability bias or differential reporting bias. Social desirability bias for sensitive behaviors such as transactional sex has the potential to attenuate some associations. The weak statistical robustness precludes further prioritization or refinement of DREAMS criteria from this analysis. The risk factors for HIV acquisition among AGYW reported in this study are similar to those reported in studies conducted in other countries in Africa [20,21]. These findings prove that the DREAMS criteria are relevant for identifying AGYW who should be enrolled in the program.

Examination of the prevalence of DREAMS criteria individually and jointly, along with the estimated proportion reached with DREAMS screening, suggests that screening efficiency has improved throughout PHN delivery of DREAMS services, with 71% of screened individuals being eligible for DREAMS services under the current screening eligibility criteria and outreach efforts. The DREAMS entry point of “other” had the highest proportion of screen-eligible individuals. The DREAMS entry points within this “other” group must be better described to inform future efforts to improve efficiency. The screening reach estimates suggest that PHN has reached AGYW 10–14 and 15–19-year-olds in their catchment several times over. Only orphans in Khomas did not reach above 100% in 10–14-year-olds. Among 15–19-year-olds, the lowest screening reach was among out-of-school girls, a criterion shown to be strongly associated with behaviorally acquired HIV in unadjusted analyses of PHIA. Among 20–24-year-olds, screening coverage did not exceed the population size estimation, and the lowest screening coverage was observed in AGYW reporting alcohol misuse (22%) and those reporting transactional sex (31%). Transactional sex was the only variable with responses robust enough to be associated with behaviorally acquired HIV. The excess screening reach may be attributed to population mobility, retesting, or inaccuracies in the denominator used, since there was no consideration of population growth. As noted in Figure 2, 1879 individuals among the 110,582 screened individuals had equal to or more than two observations. Further interrogation of the denominator-derived data and assumptions made in deriving them is required.

## 5. Strengths and Limitations

There are several strengths to this analysis. NamPHIA, a population-based assessment designed to provide national and regional estimates of the HIV care cascade, was conducted in 2017, just before PHN-DREAMS implementation started; thus, the population-level estimates offer a reasonable “baseline” measure of DREAMS needs for all regions. Using the UpSet plots to examine individual and conjoint risk factors allowed further interrogation of AGYW’s HIV risk profile, and this understanding can be used to refine screening efforts. Lastly, our methodological approach to developing the denominators for size estimation in the reach analysis has produced consistent findings with other PHIA analyses and other analyses using Namibia census data.

There are several limitations to this analysis as well. First, PHIA was conducted as a household-based survey, and individuals in a household were interviewed in succession, which may have led to social desirability bias. We did not account for this population growth, which would result in a slight underestimation of the population size throughout the implementation period of 2018–2023 and an overestimation of reach. The estimation of HIV outcomes adjusted for perinatal transmission reflects historical experience with health services and epidemiologic assumptions that might have misestimated HIV outcomes. Additionally, there might have been a misclassification bias in perinatal cases because the definition was based on never having had sex and never having experienced sexual violence, yet some participants might have been unwilling to disclose their sexual history. Due to the retrospective nature of these data, we were not able to examine the time sequence of the history of pregnancy, no/irregular condom use, and out-of-school status.

## 6. Conclusions

This analysis examined the PHN approach to reach AGYW in three regions in Namibia using DREAMS services. We observed that the PHN reach in screening far exceeded AGYW for 10–14-year-olds, saturated and exceeded in varying levels for those 15–19 years old, and the screening reach was lowest in 20–24-year-olds. We observed that screening efficiency increased throughout PHN’s implementation. However, the excess reach needs to be interpreted with caution since this may have been due to population mobility, retesting, or inaccuracies in calculating the denominator. Based on these findings, we recommend the development of a retention and re-engagement plan for sustaining AGYW 10–14 years who are at increased risk of initiating sex, falling pregnant, and not being in school. In 15–19-year-olds, PHN efforts should interrogate geographic variability, outreach, and screening practices. AGYW engaged in transactional sex should be prioritized, given the very low screening reach and association with HIV.

## Figures and Tables

**Table 1:** PEPFAR-approved eligibility criteria.

10–14 Years	15–19 Years	20–24 Years
Ever had sex History of pregnancy Experience of sexual violence (lifetime) Experience of physical or emotional violence (within the last year) Alcohol or other substance use Out of school (dropped out or never been to school) Single or double orphan	Multiple sexual partners (in the last year) History of pregnancy STI (diagnosed or treated) Transactional sex (including staying in a relationship for material or financial support) Alcohol or other substance misuse (within the last year) Single or double orphan	Multiple sexual partners (in the last year) STI (diagnosed or treated) No or irregular condom use Transactional sex (including staying in a relationship for material or financial support) Experience of sexual violence (lifetime) Alcohol or other substance misuse (within the last year)

**Table 2:** Mapping PHIA to DREAMS criteria.

DREAMS Indicator	PHIA Variable—Child	PHIA Variable—Adult	Note: Among Individuals
Out of School	adensch	schlcur, schlat	Everyone
Orphanhood	momalive, dadalive	momalive, dadalive	Everyone
Ever had sex	adhdsx	sexever	Everyone
Alcohol Misuse	X	alcfreq, alcnumday, alcsixmore	Everyone
Sexual Violence	X	prssxtimes, frcsxtimes	Everyone
History of Pregnancy	X	pregnum, sexever	are known to have ever had sex
No/Irregular Condom Use	X	partlastcndm1, partlastcndm2, partlastcndm3, sex12months, sexever	are known to have ever had sex
Transactional Sex	X	partlastsup1, partlastsup2, partlastsup3, sellsxever	are known to have ever had sex
Multiple Sex Partners	X	sexever	ever had sex

X—No variable.

## Data Availability

The original contributions presented in this study are included in the article/Supplementary Material. The dataset associated with this manuscript can be directed to the corresponding author.

## Supplementary Materials

The following supporting information can be downloaded at: https://www.mdpi.com/article/10.3390/tropicalmed10090240/s1, Figure S1: PHN programmatic reach of ever-pregnant 15–19-year-olds; Figure S2: PHN programmatic reach of 20–24-year-olds engaged in transactional sex.
