# Correction: Moyo et al. Profiling HIV Risk and Determined, Resilient, Empowered AIDS-Free, Mentored, and Safe (DREAMS) Program Reach Among Adolescent Girls and Young Women (AGYW) in Namibia: Secondary Analysis of Population and Program Data. *Trop. Med. Infect. Dis.* 2025, *10*, 240

**DOI:** 10.3390/tropicalmed11080209

**Published:** 2026-07-24

**Authors:** Enos Moyo, Endalkachew Melese, Hadrian Mangwana, Simon Takawira, Rosalia Indongo, Bernadette Harases, Perseverance Moyo, Ntombizodwa Makurira Nyoni, Kopano Robert, Tafadzwa Dzinamarira

**Affiliations:** 1 School of Nursing & Public Health, College of Health Sciences, University of Kwa-Zulu Natal, Durban 4041, South Africa; zhua2007@gmail.com; 2 Project HOPE—The People-to-People Health Foundation, Inc., Windhoek 10005, Namibia; emelese@projecthope.org; 3 Project HOPE Namibia, Windhoek 10005, Namibia; haddi81@gmail.com (H.M.); simontakawira11@gmail.com (S.T.); rindongo@projecthope.org (R.I.); bharases@projecthope.org (B.H.); 4 Clinical Department, Medical Centre Oshakati, Oshakati 15001, Namibia; moyoperseverance@gmail.com; 5 School of Nursing, Faculty of Health, Humanities, & Social Sciences, Welwitschia University, Windhoek 10005, Namibia; rkpone2000@gmail.com; 6 School of Health Systems and Public Health, Faculty of Health Sciences, University of Pretoria, Pretoria 0001, South Africa; u19385419@up.ac.za

In the original publication [1], the Acknowledgments section was not included: “This study builds on prior internal analytical work conducted under the PHN DREAMS programme, including consultancy-supported analyses. These materials were used to inform aspects of the study design and interpretation.” The authors state that the scientific conclusions are unaffected. This correction was approved by the Academic Editor. The original publication has also been updated.
