# Supplementary material for: Profiling HIV Risk and Determined, Resilient, Empowered AIDS-Free, Mentored, and Safe (DREAMS) Program Reach Among Adolescent Girls and Young Women (AGYW) in Namibia: Secondary Analysis of Population and Program Data
Source: Trop Med Infect Dis. 2025 Aug 27;10(9):240. doi: 10.3390/tropicalmed10090240 (PMC12474173; doi:10.3390/tropicalmed10090240)

Supplementary Figure S1: PHN programmatic reach of ever-pregnant 15-19-year-olds.

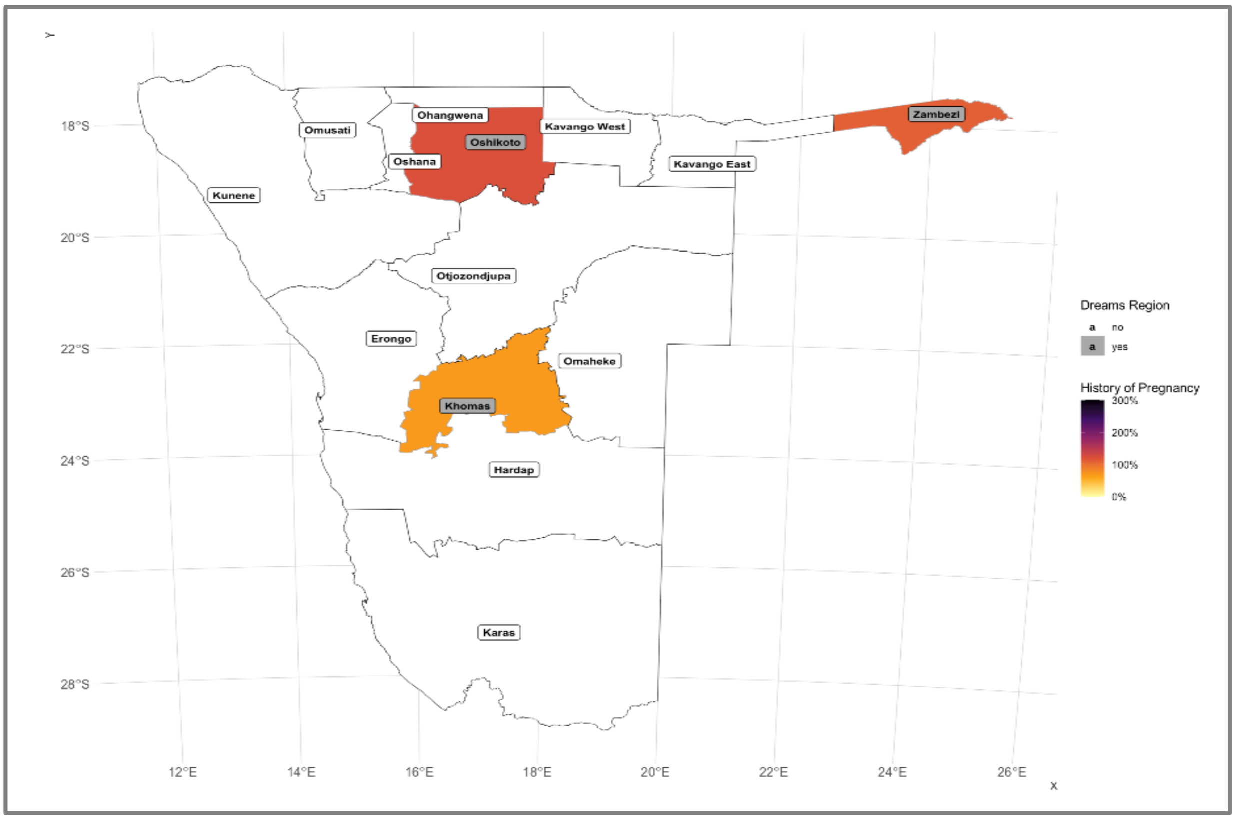

Supplementary Figure S2: PHN programmatic reach of 20-24-year-olds engaged in transactional sex.

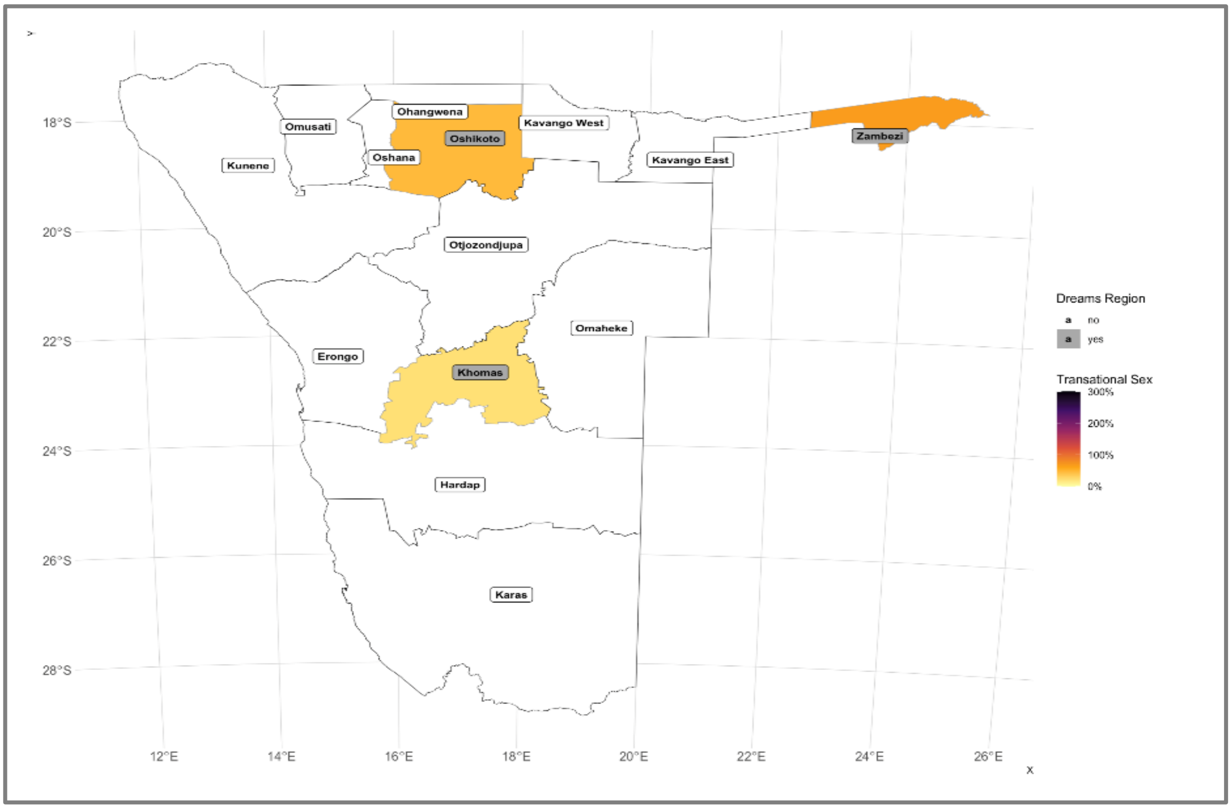

Supplement: Supplementary file 1 [file tropicalmed-10-00240-s001.zip › tropicalmed-3779930-supplementary.pdf]
